# Genetically predicted childhood body mass index and lung cancer susceptibility: A two‐sample Mendelian randomization study

**DOI:** 10.1002/cam4.6406

**Published:** 2023-08-07

**Authors:** Dongsheng Wu, Jian Zhou, Yuchen Huang, Quan Zheng, Tengyong Wang, Lunxu Liu

**Affiliations:** ^1^ Department of Thoracic Surgery and Institute of Thoracic Oncology, West China Hospital Sichuan University Chengdu China; ^2^ West China School of Medicine Sichuan University Chengdu China

**Keywords:** body mass index, childhood, lung cancer, Mendelian randomization

## Abstract

**Background:**

The association between adult body mass index (BMI) and lung cancer (LC) susceptibility have been reported, but the causal relationship with childhood BMI remains largely unclear. To evaluate the causal effect of childhood BMI on LC susceptibility, a two‐sample Mendelian randomization (MR) study was performed.

**Methods:**

The two‐sample MR analysis utilized 25 single nucleotide polymorphisms (SNPs) as instrumental variables for childhood BMI. Genetic summary data from the International Lung Cancer Consortium and FinnGen databases were analyzed to estimate the causal effect of these SNPs on LC susceptibility. The IVW method was employed as the primary analysis, supplemented by the Weighted Median, MR‐Egger, and MR pleiotropy residual sum and outlier test.

**Results:**

Our findings indicated that there was no causal association between childhood BMI and the susceptibility of LC (odds ratio [OR]: 1.03, 95% confidence interval [CI]: 0.90–1.17, *p* = 0.705), lung adenocarcinoma (OR: 0.99, 95% CI: 0.86–1.13, *p* = 0.832), lung squamous cell carcinoma (OR: 0.97, 95% CI: 0.84–1.13, *p* = 0.726), and small cell LC (OR: 1.09, 95% CI: 0.82–1.45, *p* = 0.554) based on the IVW as well as other methods employed. Furthermore, these findings indicated no causal effect of childhood BMI on the LC susceptibility in both ever smokers and never smokers.

**Conclusion:**

This study did not conclude a causal effect between childhood BMI and LC susceptibility. However, given the complex nature of cancer development, further studies are needed to verify these findings.

## 1 INTRODUCTION

Lung cancer (LC) remains the leading cause of cancer‐related deaths worldwide.^1^ While various risk factors, such as age, family history, and air pollution, have been studie,^2^, ^3^, ^4^ they are not easily modified or controllable as effective surveillance strategies. Consequently, there is a critical need to identify other modifiable risk factors to enhance individualized prevention strategies and alleviate the global burden of LC.

Body mass index (BMI) has been widely used as an indicator for assessing body fat status and evaluating the risk of diseases in both clinical and research settings, such as coronary artery disease, diabetes, and certain malignant tumors.^5^, ^6^, ^7^ The impact of adult BMI on LC susceptibility has been widely studied, with several studies indicating that higher adult BMI is associate with a reduced risk of LC.^8^, ^9^, ^10^, ^11^ However, the causal relationship between childhood BMI and LC susceptibility requires further exploration, as conducting large‐scale and long‐term randomized controlled trials poses significant challenges. Additionally, the presence of reverse causal effects and potential confounding factors, such as smoking and drinking,^12^, ^13^ can make it difficult to draw conclusive results from traditional retrospective studies.

To overcome these issues, we employed a novel epidemiology method named Mendelian randomization (MR) that reduces bias while saving time and cost.^14^ In the MR study, genetic variations such as single nucleotide polymorphisms (SNPs) are used as instrumental variables (IVs) to alternate the studied exposure. Due to their random assignment and occurrence prior to disease onset, genetic variants are largely independent of acquired or environmental factors.^15^ This allows MR to assess the causal effect of childhood BMI on LC susceptibility while avoiding biases caused by reverse causal effects or confounding factors. So far, one MR analysis related to this topic was reported.^16^ However, this study only examined potential causal relationships using a relatively small sample size, and it did not fully explore other LC subgroups, such as detailed histologic subtypes and LC with different smoking statuses,^16^ which warranted further exploration.

In this context, this MR study combined large‐size GWAS data to address these gaps by thoroughly investigating the potential causal associations between childhood BMI and LC susceptibility, specifically focusing on various LC subgroups.

## 2 METHODS

### 2.1 Study design

**FIGURE 1:**
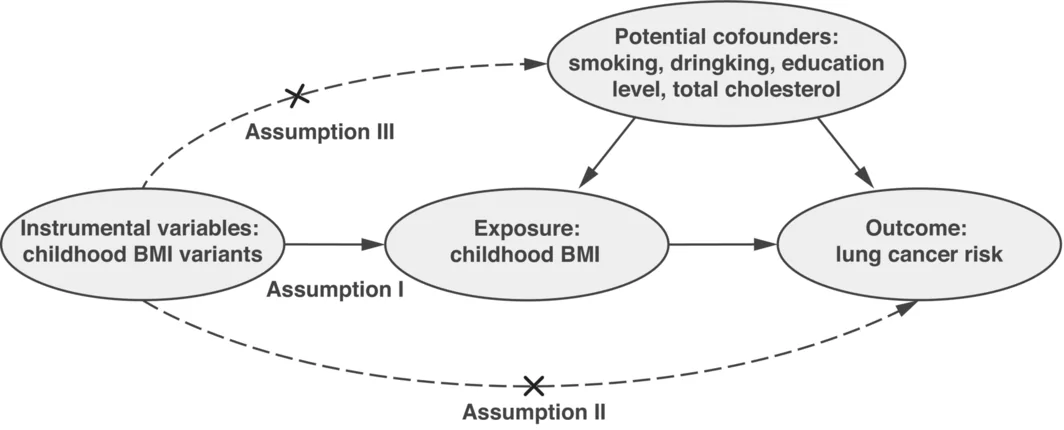
Schematic illustration depicted Mendelian randomization assumptions. The assumptions including: (I) genetic instruments are significantly associated childhood BMI; (II) genetic instruments can only influence lung cancer through childhood BMI without any alternative causal pathways; (III) genetic instruments are independent of any cofounder.

We conducted the two‐sample the two‐sample MR analysis using GWAS summary level data obtained from recently published GWAS meta‐analyses and public database. The GWAS summary level data for the exposure and outcome were obtained from separate samples to ensure that research participants did not overlap. MR study adheres to three principal assumptions as follows: (i) the genetic instruments are associated with childhood BMI; (ii) the genetic instruments can only affect LC through childhood BMI without any other causal pathway; and (iii) the genetic instruments are not affected by any potential confounder,^17^ which are depicted in Figure 1. No ethical approval was required for the study since it involved a re‐analysis of the published data.

### 2.2 Genetic variants for childhood BMI

We retrieved the GWAS summary level data for childhood BMI from the recently published GWAS study by the Early Growth Genetics consortium (EGG), which combined 41 studies enrolling 61,111 children aged 2–10 years in European ancestry.^18^ All parameters, including height and weight, were measured using standard international unit and BMI was calculated by dividing weight by the square of height (kg/m^2^). Genetic instruments with significant genome‐wide significance of *p* < 5 × 10^−8^ were included, which were then clumped using the window of 500 kb and linkage disequilibrium of *r* ^2^ less than 0.2 to ensure that the variants were independent.^18^ SNPs that were not found in the outcome GWAS summary data or had palindrome structures were removed for a low loss proportion during matching and harmonizing. Finally, the instrumental variables consisted of 25 single‐nucleotide polymorphisms (SNPs), which was demonstrated significantly associated with childhood BMI. Weak instruments resulted in inadequate ability to predict causal effect. Therefore, we calculated explained genetic variation (*R* ^2^) and *F* statistic to evaluate these instruments. The former was generated using the formula: *R* ^2^ = 2 × *β* ^2^ × EAF × (1 − EAF), with *β* being estimated genetic effect of each SNP and EAF being the effect allele frequency, while the latter was calculated using formula: $F=\left(\frac{N-1-K}{K}\right)\left(\frac{R^{2}}{1-R^{2}}\right)$, with *N* being sample size and *K* being number of SNPs.^19^ The *F* statistic values, more than a conventional threshold of 10, indicating sufficient strength to predict childhood BMI.^20^

### 2.3 Genetic variants for LC

We obtained GWAS summary level data for LC from two databases, namely the International Lung Cancer Consortium (ILCCO),^21^ with data updated in 2021, and FinnGen (www.finbb.fi), utilizing the latest version 9 for this study. The LC phenotype was defined as a binary trait, encompassing 29,266 LC cases and 56,450 controls from ILCCO, along with 5842 LC cases and 281,295 controls from FinnGen (Table S1).

### 2.4 Genetic variants for risk factors of LC

To explore whether childhood BMI potentially affected LC susceptibility through other risk factors, we performed inverse‐variance weighted (IVW) analysis to assess the association between childhood BMI and these factors. Based on existing literature and available GWAS data,^12^, ^22^ we included four potential risk factors in our study, including smoking status, drinking status, education level, and total cholesterol. GWAS summary level data for smoking status (age of smoking initiation, ever vs. current smoker, ever vs. never smoker, and cigarettes smoked per day) and alcohol consumption (drinks per week) were obtained from the GWAS & Sequencing Consortium of Alcohol and Nicotine use (GSCAN).^23^ The GWAS data for education level were obtained from the Social Science Genetic Association Consortium (SSGAC).^24^ We evaluated the relationship of childhood BMI on total cholesterol using data from the Global Lipids Genetics Consortium (GLGC).^25^ The information of GWAS summary data is presented in Table S1.

### 2.5 Mendelian randomization

The MR analyses were conducted independently within each outcome database, followed by a fixed‐effect meta‐analysis to combine the casual estimates. Several MR methods were employed to explore the potential causal impact of childhood BMI on LC susceptibility. First, we applied the IVW to estimate the causal impact of childhood BMI on LC susceptibility, which weights the regression model using the inverse of the outcome variance (the square of the standard error) and ignores the intercept of the regression model.^26^ Notably, when using IVW method, the existence of the horizontal pleiotropy may result in biased result. Therefore, we performed MR–Egger regression and weighted‐median method to complement the IVW method. MR–Egger regression is similar to IVW method, otherwise, which allows the presence of horizontal pleiotropy.^27^ When dealing with invalid instruments, the weighted‐median method is more robust MR approach than IVW and MR–Egger regression.^28^ Furthermore, we tested the horizontal pleiotropy using MR pleiotropy residual sum and outlier (MR‐PRESSO) method and re‐calculate the causal effect after removing outliers.^29^ A two‐tailed *p* value <0.05 was considered significant. We conducted all MR analyses in R software (version 4.2.1) utilizing the packages “MRPRESSO” (version 1.0), “TwoSampleMR” (version 0.5.6), and “meta” (version 3.1.1).

### 2.6 Sensitivity analysis

Several sensitivity analyses, including the pleiotropy test, heterogeneity test, and leave‐one‐out sensitivity test, were conducted to examine the robustness of the results. The horizontal pleiotropic effects were examined using intercept test of MR‐Egger. Heterogeneity was measured by Cochran's Q statistic and *I* ^2^. Furthermore, leave‐one‐out analysis was performed to evaluate the potential impact of individual SNPs on the IVW estimate by removing one SNP every time. The power analysis for our study was conducted employing the mRnd power calculator (https://shiny.cnsgenomics.com/mRnd/).^30^

## 3 RESULTS

The genetic instruments explained 2.95% for childhood BMI and F statistic values ranged from 36 to 153, indicating strong instruments. The detailed information of SNPs is summarized in Table S2.

### 3.1 Causal effect of childhood BMI on LC susceptibility

**FIGURE 2:**
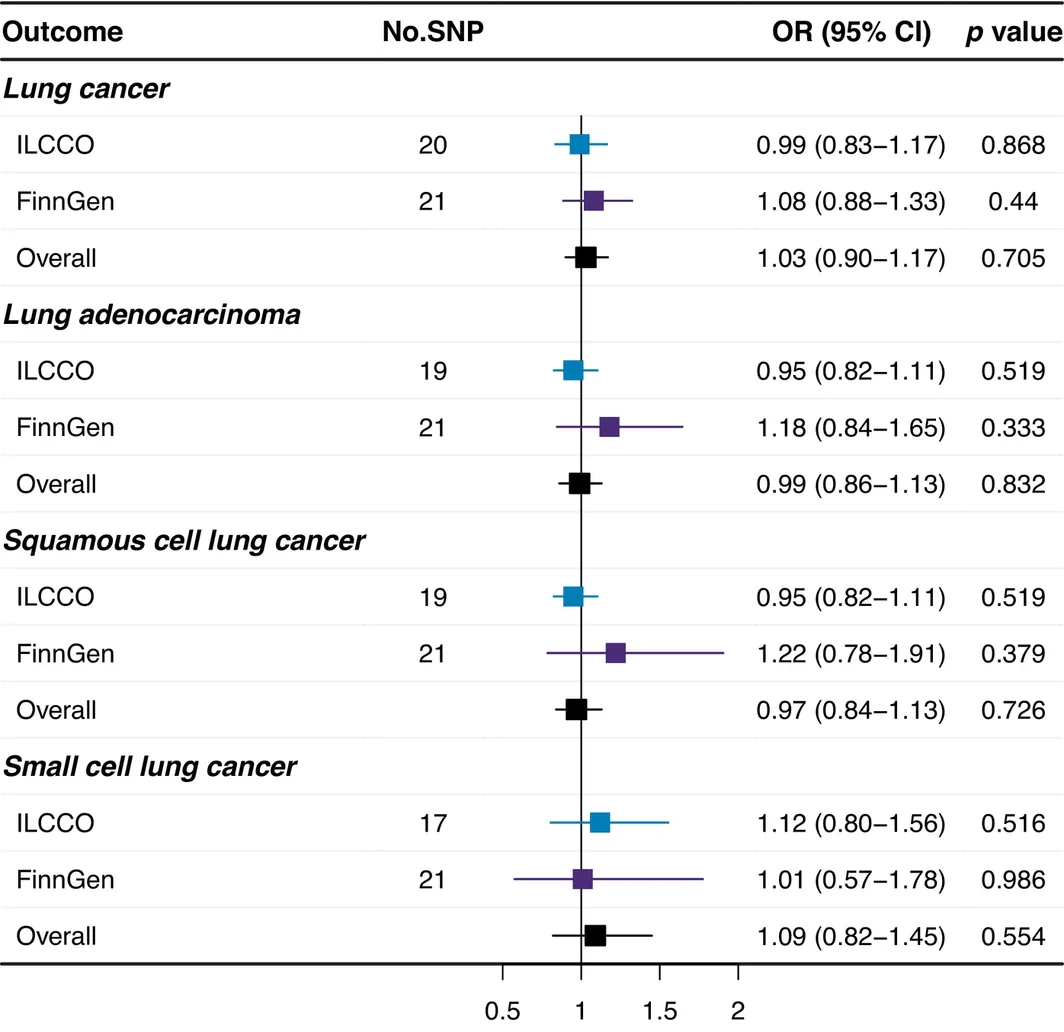
Mendelian randomization using IVW method estimated the causal effects between childhood BMI and lung cancer susceptibility.

The MR estimates in each outcome database in line with the meta‐analysis showed consistent results. In the meta‐analysis, the MR study using the IVW did not reveal a causal effect between childhood BMI and LC (odds ratio [OR]: 1.03, 95% confidence interval [CI]: 0.90–1.17, *p* = 0.705) (Figure 2). The results of the MR–Egger regression, weighted median, and MR‐PRESSO analysis were in line with those of the IVW (Table S3). Similar results of IVW were also observed in different LC subgroups, including lung adenocarcinoma (OR: 0.99, 95% CI: 0.86–1.13, *p* = 0.832), lung squamous cell carcinoma (OR: 0.97, 95% CI: 0.84–1.13, *p* = 0.726), small cell lung cancer (SCLC) (OR: 1.09, 95% CI: 0.82–1.45, *p* = 0.554), and LC in both ever smoker (OR: 0.90, 95% CI: 0.74–1.11, *p* = 0.329) and never smoker (OR: 0.93, 95% CI: 0.68–1.28, *p* = 0.674), as well as other methods (Figure 2; Figure S1 and S2; Table S3).

### 3.2 Sensitivity and pleiotropy analysis

Regarding the heterogeneity analysis, we observed significant heterogeneity in the causal effect analysis of childhood BMI on susceptibility of LC, as well as LC in ever smokers (*p* < 0.001 in both the MR‐Egger and IVW) using outcome data from ILCCO. However, no significant heterogeneity was found in the other LC subgroups (*p* > 0.05 in both the Egger and IVW) (Table S4). Nevertheless, the causal effect of childhood BMI on susceptibility of LC, as well as LC in ever smokers, was consistent in direction and magnitude across different methods, indicating a robust result. Furthermore, no evidence of horizontal pleiotropy in other groups was found through MR–Egger regression analysis (Table S4). In the leave‐one‐out analysis, we did not identify any individual SNP that had a strong impact on the causal estimate (Figure S3 and S4), and the funnel plot revealed no evidence of bias in the instrumental variables (Figure S5 and S6).

### 3.3 Causal relationship of childhood BMI on risk factors

We performed additional MR analyses using the IVW method to estimate the causal relationship between childhood BMI and potential risk factors of LC. The results revealed that each 1 kg/m^2^ increase in childhood BMI was associated with a 6% decrease in the likelihood of ever smoking (OR: 0.94, 95% CI: 0.90–0.98, *p* = 0.006) and a decrease in smoking intensity (OR: 0.94, 95% CI: 0.90–0.98, *p* = 0.003). Nevertheless, the findings did not demonstrate significant causal effects between childhood BMI and the age of smoking initiation, smoking cessation, drinking per week, years of education, and total cholesterol (Table S5).

## 4 DISCUSSION

In this two‐sample MR study, we investigated the causal association between childhood BMI and LC susceptibility. The results did not support a causal effect of genetically predicted childhood BMI on LC susceptibility or any of the examined LC subgroups. These findings remained robust when considering pleiotropy and heterogeneity. Additionally, the consistency of results obtained from the Weighted median, MR‐Egger, and IVW analyses further supported the robustness of these findings.

To our knowledge, one MR study investigated the correlation between childhood BMI and LC susceptibility, and our results aligned with its findings.^16^ Compared with the current study, the study by Gao et al.^16^ used GWAS summary level data for LC with relatively small sample size (12,160 cases and 16,838), which may limit the evidence power. In this study, we obtained large sample size GWAS summary data from both currently largest meta‐analysis for LC and FinnGen database. Also, a fixed‐effect meta‐analysis was employed to combine the casual effects from the two outcome databases, which provided more convinced conclusion. Second, the study conducted by Gao et al.^16^ did not fully explore certain LC subgroups, such as detailed histologic subtypes and LC with different smoking statuses, which was further explored in this study. Our results provided renewed evidence that childhood BMI did not have a direct causal relationship with either SCLC or non‐small cell lung cancer (NSCLC). Although we did not observe a significant causality, our MR analysis revealed that high childhood BMI might tend to be associated with an increased risk of SCLC, but a reduced risk of NSCLC. The difference in outcomes may be attributed to the inherent heterogeneity in the impact of childhood BMI on different LC subtypes or may be influenced by the relatively small sample size of SCLC.

Our findings suggested that no apparent causal relationship existed between childhood BMI and LC susceptibility. However, abnormal childhood BMI may still influence the morbidity of LC beyond the scope of our analysis. This is because adipose, considered as an active endocrine organ, can secrete various adipokines and interleukins, such as adiponectin and TNF‐α, which may drive chronic inflammation and the subsequent development of cancer.^31^ Additionally, excessive adipose tissue can cause immune cell dysfunction and facilitate the formation of tumor‐promoting environment.^32^ Meanwhile, growing clinical research has reported that abnormal BMI during adolescence may contribute to increased risk of several malignancies in adulthood, such as breast cancer and primary hepatic cancer.^16^, ^33^ And in a 50‐year cohort study, it was found that per standard deviation increase in childhood BMI could increase the overall risk of smoking‐related cancer by 30%.^34^ However, this study did not provide the detailed results in each cancer type, which precluded the establishment of a causal correlation between childhood BMI and smoking‐related LC. In this study, our results suggested that there existed little causal effect between childhood BMI and susceptibility of LC in ever and current smokers. While BMI is a useful tool for evaluating body fat, other indicators, for example waist‐to‐hip ratio, are also worth exploring. Combining BMI with waist‐to‐hip ratio or other indicators to assess the impact of childhood BMI on risk of LC may have promising clinical significance.

It was worth noting that prior study have established a correlation between smoking status and BMI, whereby smoking is associated with weight loss, while smoking cessation often leads to weight gain.^35^ These relationships can be explained through various mechanisms such as changes in neural pathways^36^ and gut microbiota.^37^ Interestingly, in our analysis, we observed a causal relationship between increasing childhood BMI and a decreased likelihood of smoking. This finding underscores the complex interplay between smoking status and BMI. It highlights the need for further research to elucidate the underlying mechanisms driving this association.

Our study presents several strengths and practical implications. First, we adopted MR analysis to stimulate randomized controlled trails, which offers several advantages compared to traditional retrospective studies. For instance, this innovative method conserves physical, and financial resources, while also reducing potential confounding biases and reverse causal effects. Second, given the heavy burdens of LC and childhood obesity globally, revealing potential causality between childhood BMI and LC incidence may influence the future health care policymaking. These results may also provide valuable insights into the ideal timing for disease intervention, thereby contributing to the overall efforts aimed at reducing the burden of these two critical health issues.

However, as with any MR analysis, limitations are inevitable. First, the generalizability of our conclusions to other populations remains uncertain, as the participants included in our study were exclusively of European ancestry. Second, despite our efforts to combine available data, a larger sample size in the GWAS data would have further enhanced the statistical power of our MR analysis. In addition, BMI is intrinsically affected by the interactions of congenital and environmental factors such as dietary habits.^12^ Therefore, our study is constrained within the topic of genetically predicted childhood BMI. Regarding no positive evidence has been found in this current study, more explorations are needed to investigate the relation between environmentally determined childhood BMI and LC susceptibility.^38^

## 5 CONCLUSION

Our study provided the latest evidence indicating the absence of a causal relationship between childhood BMI and LC susceptibility. Therefore, utilizing childhood BMI as a screening tool for assessing LC susceptibility may not yield effective results. Instead, greater emphasis should be placed on uncovering the relationship between childhood BMI influenced by environmental factors such as dietary habits and LC susceptibility.

## AUTHOR CONTRIBUTIONS

**Dongsheng Wu:** Investigation (equal); methodology (lead); visualization (equal); writing – review and editing (equal). **Jian Zhou:** Funding acquisition (supporting); investigation (lead); visualization (equal); writing – review and editing (equal). **Yuchen Huang:** Investigation (equal); methodology (lead); visualization (equal); writing – review and editing (equal). **Quan Zheng:** Investigation (equal); writing – review and editing (equal). **Tengyong Wang:** Investigation (equal); writing – review and editing (equal). **Lunxu Liu:** Conceptualization (lead); supervision (lead); writing – review and editing (lead).

## FUNDING INFORMATION

This study was supported by 1.3.5 Project for Disciplines of Excellence, West China Hospital, Sichuan University (Grant number: ZYGD18021 to Dr. Lunxu Liu) and National Natural Science Foundation of China (Grant number: 82102968 to Dr. Jian Zhou).

## CONFLICT OF INTEREST STATEMENT

There is no conflict of interest to declare by the authors.

## ETHICS STATEMENT

No ethical approval was required for the study since it involved a re‐analysis of published data.

## Supporting information


Figure S1.
Figure S2.
Figure S3.
Figure S4.
Figure S5.
Figure S6.

Table S1.
Table S2.
Table S3.
Table S4.
Table S5.

## Data Availability

All GWAS summary data included in this study are publicly available.

## References

[1] Sung H , Ferlay J , Siegel RL , et al. Global cancer statistics 2020: GLOBOCAN estimates of incidence and mortality worldwide for 36 cancers in 185 countries. CA Cancer J Clin. 2021;71(3):209‐249. 33538338 10.3322/caac.21660

[2] Wood DE , Kazerooni EA , Aberle D , et al. NCCN guidelines(R) insights: lung cancer screening, version 1.2022. J Natl Compr Canc Netw. 2022;20(7):754‐764. 35830884 10.6004/jnccn.2022.0036

[3] Ang L , Chan CPY , Yau WP , Seow WJ . Association between family history of lung cancer and lung cancer risk: a systematic review and meta‐analysis. Lung Cancer. 2020;148:129‐137. 32892102 10.1016/j.lungcan.2020.08.012

[4] Liang H , Zhou X , Zhu Y , et al. Association of outdoor air pollution, lifestyle, genetic factors with the risk of lung cancer: a prospective cohort study. Environ Res. 2023;218:114996. 36481370 10.1016/j.envres.2022.114996

[5] Geng T , Smith CE , Li C , Huang T . Childhood BMI and adult type 2 diabetes, coronary artery diseases, chronic kidney disease, and cardiometabolic traits: a Mendelian randomization analysis. Diabetes Care. 2018;41(5):1089‐1096. 29483184 10.2337/dc17-2141

[6] Petrelli F , Cortellini A , Indini A , et al. Association of obesity with survival outcomes in patients with cancer: a systematic review and meta‐analysis. JAMA Netw Open. 2021;4(3):e213520. 33779745 10.1001/jamanetworkopen.2021.3520 PMC8008284

[7] Picon‐Ruiz M , Morata‐Tarifa C , Valle‐Goffin JJ , Friedman ER , Slingerland JM . Obesity and adverse breast cancer risk and outcome: mechanistic insights and strategies for intervention. CA Cancer J Clin. 2017;67(5):378‐397. 28763097 10.3322/caac.21405 PMC5591063

[8] Renehan AG , Tyson M , Egger M , Heller RF , Zwahlen M . Body‐mass index and incidence of cancer: a systematic review and meta‐analysis of prospective observational studies. Lancet. 2008;371(9612):569‐578. 18280327 10.1016/S0140-6736(08)60269-X

[9] You D , Wang D , Wu Y , et al. Associations of genetic risk, BMI trajectories, and the risk of non‐small cell lung cancer: a population‐based cohort study. BMC Med. 2022;20(1):203. 35658861 10.1186/s12916-022-02400-6 PMC9169327

[10] Smith L , Brinton LA , Spitz MR , et al. Body mass index and risk of lung cancer among never, former, and current smokers. J Natl Cancer Inst. 2012;104(10):778‐789. 22457475 10.1093/jnci/djs179 PMC3352831

[11] Yu D , Zheng W , Johansson M , et al. Overall and central obesity and risk of lung cancer: a pooled analysis. J Natl Cancer Inst. 2018;110(8):831‐842. 29518203 10.1093/jnci/djx286 PMC6093439

[12] Zhou H , Zhang Y , Liu J , et al. Education and lung cancer: a Mendelian randomization study. Int J Epidemiol. 2019;48(3):743‐750. 31219597 10.1093/ije/dyz121

[13] Chen C , Hu Q , Wang J , et al. Habitual consumption of alcohol with meals and lung cancer: a Mendelian randomization study. Ann Transl Med. 2021;9(3):263. 33708890 10.21037/atm-20-3063 PMC7940946

[14] Burgess S , Scott RA , Timpson NJ , Davey Smith G , Thompson SG . Using published data in Mendelian randomization: a blueprint for efficient identification of causal risk factors. Eur J Epidemiol. 2015;30(7):543‐552. 25773750 10.1007/s10654-015-0011-z PMC4516908

[15] Lawlor DA , Harbord RM , Sterne JAC , Timpson N , Davey SG . Mendelian randomization: using genes as instruments for making causal inferences in epidemiology. Stat Med. 2008;27(8):1133‐1163. 17886233 10.1002/sim.3034

[16] Gao C , Patel CJ , Michailidou K , et al. Mendelian randomization study of adiposity‐related traits and risk of breast, ovarian, prostate, lung and colorectal cancer. Int J Epidemiol. 2016;45(3):896‐908. 27427428 10.1093/ije/dyw129 PMC6372135

[17] VanderWeele TJ , Tchetgen EJT , Cornelis M , Kraft P . Methodological challenges in mendelian randomization. Epidemiology. 2014;25(3):427‐435. 24681576 10.1097/EDE.0000000000000081 PMC3981897

[18] Vogelezang S , Bradfield JP , Ahluwalia TS , et al. Novel loci for childhood body mass index and shared heritability with adult cardiometabolic traits. PLoS Genet. 2020;16(10):e1008718. 33045005 10.1371/journal.pgen.1008718 PMC7581004

[19] Palmer TM , Lawlor DA , Harbord RM , et al. Using multiple genetic variants as instrumental variables for modifiable risk factors. Stat Methods Med Res. 2012;21(3):223‐242. 21216802 10.1177/0962280210394459 PMC3917707

[20] Holsinger KE , Weir BS . Genetics in geographically structured populations: defining, estimating and interpreting F (ST). Nat Rev Genet. 2009;10(9):639‐650. 19687804 10.1038/nrg2611 PMC4687486

[21] McKay JD , Hung RJ , Han Y , et al. Large‐scale association analysis identifies new lung cancer susceptibility loci and heterogeneity in genetic susceptibility across histological subtypes. Nat Genet. 2017;49(7):1126‐1132. 28604730 10.1038/ng.3892 PMC5510465

[22] Larsson SC , Carter P , Kar S , et al. Smoking, alcohol consumption, and cancer: a mendelian randomisation study in UK biobank and international genetic consortia participants. PLoS Med. 2020;17(7):e1003178. 32701947 10.1371/journal.pmed.1003178 PMC7377370

[23] Liu M , Jiang Y , Wedow R , et al. Association studies of up to 1.2 million individuals yield new insights into the genetic etiology of tobacco and alcohol use. Nat Genet. 2019;51(2):237‐244. 30643251 10.1038/s41588-018-0307-5 PMC6358542

[24] Okbay A , Beauchamp JP , Fontana MA , et al. Genome‐wide association study identifies 74 loci associated with educational attainment. Nature. 2016;533(7604):539‐542. 27225129 10.1038/nature17671 PMC4883595

[25] Willer CJ , Schmidt EM , Sengupta S , et al. Discovery and refinement of loci associated with lipid levels. Nat Genet. 2013;45(11):1274‐1283. 24097068 10.1038/ng.2797 PMC3838666

[26] Burgess S , Butterworth A , Thompson SG . Mendelian randomization analysis with multiple genetic variants using summarized data. Genet Epidemiol. 2013;37(7):658‐665. 24114802 10.1002/gepi.21758 PMC4377079

[27] Bowden J , Davey Smith G , Burgess S . Mendelian randomization with invalid instruments: effect estimation and bias detection through Egger regression. Int J Epidemiol. 2015;44(2):512‐525. 26050253 10.1093/ije/dyv080 PMC4469799

[28] Bowden J , Davey Smith G , Haycock PC , Burgess S . Consistent estimation in Mendelian randomization with some invalid instruments using a weighted median estimator. Genet Epidemiol. 2016;40(4):304‐314. 27061298 10.1002/gepi.21965 PMC4849733

[29] Verbanck M , Chen CY , Neale B , Do R . Detection of widespread horizontal pleiotropy in causal relationships inferred from Mendelian randomization between complex traits and diseases. Nat Genet. 2018;50(5):693‐698. 29686387 10.1038/s41588-018-0099-7 PMC6083837

[30] Brion MJ , Shakhbazov K , Visscher PM . Calculating statistical power in Mendelian randomization studies. Int J Epidemiol. 2013;42(5):1497‐1501. 24159078 10.1093/ije/dyt179 PMC3807619

[31] Boutari C , Mantzoros CS . Inflammation: a key player linking obesity with malignancies. Metabolism. 2018;81:A3‐A6. 29309747 10.1016/j.metabol.2017.12.015

[32] Ringel AE , Drijvers JM , Baker GJ , et al. Obesity shapes metabolism in the tumor microenvironment to suppress anti‐tumor immunity. Cell. 2020;183(7):1848‐1866.e26. 33301708 10.1016/j.cell.2020.11.009 PMC8064125

[33] Berentzen TL , Gamborg M , Holst C , Sørensen TI , Baker JL . Body mass index in childhood and adult risk of primary liver cancer. J Hepatol. 2014;60(2):325‐330. 24076363 10.1016/j.jhep.2013.09.015

[34] Jeffreys M , Smith GD , Martin RM , Frankel S , Gunnell D . Childhood body mass index and later cancer risk: a 50‐year follow‐up of the Boyd Orr study. Int J Cancer. 2004;112(2):348‐351. 15352051 10.1002/ijc.20423

[35] Aubin H‐J , Farley A , Lycett D , Lahmek P , Aveyard P . Weight gain in smokers after quitting cigarettes: meta‐analysis. BMJ. 2012;345:e4439. 22782848 10.1136/bmj.e4439 PMC3393785

[36] Calarco CA , Picciotto MR . Nicotinic acetylcholine receptor signaling in the hypothalamus: mechanisms related to Nicotine's effects on food intake. Nicotine Tob Res. 2020;22(2):152‐163. 30690485 10.1093/ntr/ntz010 PMC7297099

[37] Fluhr L , Mor U , Kolodziejczyk AA , et al. Gut microbiota modulates weight gain in mice after discontinued smoke exposure. Nature. 2021;600(7890):713‐719. 34880502 10.1038/s41586-021-04194-8

[38] Chen X , Kong J , Diao X , et al. Depression and prostate cancer risk: a Mendelian randomization study. Cancer Med. 2020;9(23):9160‐9167. 33027558 10.1002/cam4.3493 PMC7724297

