# Supplementary material for: Genetically predicted childhood body mass index and lung cancer susceptibility: A two‐sample Mendelian randomization study
Source: Cancer Med. 2023 Aug 7;12(17):18418–24. doi: 10.1002/cam4.6406 (PMC10523972; doi:10.1002/cam4.6406)
Supplement: Supplementary file 1 — Figure S1. Figure S2. Figure S3. Figure S4. Figure S5. Figure S6. [file CAM4-12-18418-s001.docx]

**Supplementary Figures**

**Figure S1. Scatter plots showing the causal association of childhood BMI on lung cancer based on ILCCO database.**

**Figure S2. Scatter plots showing the causal association of childhood BMI on lung cancer based on FinnGen database.**

**Figure S3. Leave-one-out plots for childhood BMI on lung cancer based on ILCCO database.**

**Figure S4. Leave-one-out plots for childhood BMI on lung cancer based on FinnGen database.**

**Figure S5. Funnel plots for childhood BMI on lung cancer based on ILCCO database.**

**Figure S6. Funnel plots for childhood BMI on lung cancer based on FinnGen database.**

**Figure S1.** Scatter plots showing the causal association of childhood BMI on lung cancer based on ILCCO database, with the slope of each line corresponding to estimated causal effect per method. A, overall lung cancer; B, lung adenocarcinoma; C, squamous cell lung cancer; D, small cell lung cancer; E, Lung cancer in ever smoker; F, lung cancer in never smoker.

**Figure S2.** Scatter plots showing the causal association of childhood BMI on lung cancer based on FinnGen database. A, overall lung cancer; B, lung adenocarcinoma; C, squamous cell lung cancer; D, small cell lung cancer.

**Figure S3.** Leave-one-out plots for childhood BMI on lung cancer based on ILCCO database. A, overall lung cancer; B, lung adenocarcinoma; C, squamous cell lung cancer; D, small cell lung cancer; E, lung cancer in ever smoker; F, lung cancer in never smoker.

**Figure S4.** Leave-one-out plots for childhood BMI on lung cancer based on FinnGen database. A, overall lung cancer; B, lung adenocarcinoma; C, squamous cell lung cancer; D, small cell lung cancer.

**Figure S5.** Funnel plots for childhood BMI on lung cancer based on ILCCO database. A, overall lung cancer; B, lung adenocarcinoma; C, squamous cell lung cancer; D, small cell lung cancer; E, lung cancer in ever smoker; F, lung cancer in never smoker.

**Figure S6.** Funnel plots for childhood BMI on lung cancer based on FinnGen database. A, overall lung cancer; B, lung adenocarcinoma; C, squamous cell lung cancer; D, small cell lung cancer.
