# Supplementary material for: Genetically predicted childhood body mass index and lung cancer susceptibility: A two‐sample Mendelian randomization study
Source: Cancer Med. 2023 Aug 7;12(17):18418–24. doi: 10.1002/cam4.6406 (PMC10523972; doi:10.1002/cam4.6406)
Supplement: Supplementary file 2 — Table S1. Table S2. Table S3. Table S4. Table S5. [file CAM4-12-18418-s002.docx]

**Supplementary Tables**

**Table S1. Details of GWAS data included in Mendelian randomization analyses.**

**Table S2. 25 instrument variables for childhood BMI.**

**Table S3. Effect estimates of the associations of genetic instrumental variables for Childhood BMI and risk of lung cancer.**

**Table S4. Heterogeneity and MR-Egger regression analysis of the associations between childhood BMI and lung cancer susceptibility.**

**Table S5. Causal effect of childhood BMI on risk factors of lung cancer.**

**Table S1. Details of GWAS data included in Mendelian randomization analyses.**

| **Variable** | **Consortium** | **Case/control^#^** | **Population** | **PMID** |
| --- | --- | --- | --- | --- |
| Lung cancer | ILCCO | 29,266/56,450 | European | 28604730 |
|  | FinnGen | 5,842/281,295 | European | www.finbb.fi |
| Lung adenocarcinoma | ILCCO | 11,273/55,483 | European | 28604730 |
|  | FinnGen | 1,553/285,584 | European | www.finbb.fi |
| Lung squamous cell carcinoma | ILCCO | 7,426/55,627 | European | 28604730 |
|  | FinnGen | 1,413/285,724 | European | www.finbb.fi |
| Small cell lung cancer | ILCCO | 2,664/21,444 | European | 28604730 |
|  | FinnGen | 676/286,461 | European | www.finbb.fi |
| Lung cancer in ever smoker | ILCCO | 23,223/16,964 | European | 28604730 |
| Lung cancer in never smoker | ILCCO | 2,355/7,504 | European | 28604730 |
| Smoking: ever vs. current | GSCAN | 407,766/139,453 | European | 30643251 |
| Smoking: ever vs. never | GSCAN | 557,337/674,754 | European | 30643251 |
| Childhood BMI | EGG | 61,111 | European | 33045005 |
| Age of smoking initiation | GSCAN | 341,427 | European | 30643251 |
| Cigarettes smoked per day | GSCAN | 337,334 | European | 30643251 |
| Drinking per week | GSCAN | 941,280 | European | 30643251 |
| Education level | SSGAC | 405,072 | European | 27225129 |
| Total cholesterol | GLGC | 93,982 | European | 24097068 |

Note: ^#^sample size of categorical variables was presented as case/control, while sample size of continuous variables was presented as total sample size.

BMI, body mass index; ILCCO, International Lung Cancer Consortium; GSCAN, GWAS & Sequencing Consortium of Alcohol and Nicotine use; EGG, Early Growth Genetics consortium; SSGAC, Social Science Genetic Association Consortium; GLGC, Global Lipids Genetics Consortium.

**Table S2. 25 instrument variables for childhood BMI.**

| **SNP** | **Chr** | **Position** | **EA** | **OA** | **EAF** | **Beta** | **SE** | **P value** | **Sample** | **R^2^** | **F statistic** |
| --- | --- | --- | --- | --- | --- | --- | --- | --- | --- | --- | --- |
| rs12042908 | 1 | 74997762 | A | G | 0.46 | 0.064 | 0.006 | 6.37E-25 | 61,111 | 2.03E-03 | 124.61 |
| rs543874 | 1 | 177889480 | G | A | 0.19 | 0.075 | 0.008 | 6.02E-22 | 61,111 | 1.73E-03 | 105.99 |
| rs144376234 | 1 | 110114504 | T | C | 0.04 | 0.111 | 0.017 | 1.38E-10 | 61,111 | 9.46E-04 | 57.88 |
| rs1094647 | 1 | 205655378 | G | A | 0.55 | 0.038 | 0.006 | 7.20E-10 | 61,111 | 7.15E-04 | 43.71 |
| rs61765651 | 1 | 72754314 | C | T | 0.83 | 0.047 | 0.008 | 4.99E-09 | 61,111 | 6.23E-04 | 38.12 |
| rs11676272 | 2 | 25141538 | G | A | 0.46 | 0.071 | 0.006 | 3.79E-30 | 61,111 | 2.50E-03 | 153.43 |
| rs939584 | 2 | 621558 | T | C | 0.83 | 0.092 | 0.008 | 3.73E-29 | 61,111 | 2.39E-03 | 146.31 |
| rs62107261 | 2 | 422144 | T | C | 0.95 | 0.121 | 0.018 | 9.93E-12 | 61,111 | 1.39E-03 | 85.12 |
| rs114670539 | 2 | 207064335 | T | C | 0.05 | 0.088 | 0.015 | 1.92E-09 | 61,111 | 7.36E-04 | 44.99 |
| rs12641981 | 4 | 45179883 | T | C | 0.44 | 0.045 | 0.006 | 1.29E-12 | 61,111 | 9.98E-04 | 61.04 |
| rs13107325 | 4 | 103188709 | T | C | 0.07 | 0.082 | 0.014 | 1.38E-09 | 61,111 | 8.75E-04 | 53.55 |
| rs7719067 | 5 | 153538241 | A | G | 0.43 | 0.036 | 0.006 | 6.54E-09 | 61,111 | 6.35E-04 | 38.85 |
| rs2076308 | 6 | 50791640 | C | G | 0.19 | 0.058 | 0.008 | 3.07E-13 | 61,111 | 1.04E-03 | 63.34 |
| rs116664060 | 6 | 31592524 | C | G | 0.18 | 0.049 | 0.009 | 4.63E-08 | 61,111 | 7.09E-04 | 43.34 |
| rs62500888 | 8 | 28061823 | A | G | 0.57 | 0.037 | 0.006 | 1.81E-09 | 61,111 | 6.71E-04 | 41.04 |
| rs56133711 | 11 | 27723334 | A | G | 0.24 | 0.056 | 0.007 | 3.75E-15 | 61,111 | 1.14E-03 | 69.99 |
| rs11030391 | 11 | 28644626 | A | G | 0.63 | 0.036 | 0.006 | 1.51E-08 | 61,111 | 6.04E-04 | 36.94 |
| rs11215427 | 11 | 115093438 | G | C | 0.74 | 0.039 | 0.007 | 4.64E-08 | 61,111 | 5.85E-04 | 35.79 |
| rs7138803 | 12 | 50247468 | A | G | 0.37 | 0.072 | 0.006 | 4.23E-30 | 61,111 | 2.42E-03 | 148.05 |
| rs4477562 | 13 | 54104968 | T | C | 0.13 | 0.065 | 0.009 | 5.81E-13 | 61,111 | 9.56E-04 | 58.46 |
| rs17817449 | 16 | 53813367 | G | T | 0.40 | 0.069 | 0.006 | 2.98E-27 | 61,111 | 2.29E-03 | 139.97 |
| rs114285994 | 16 | 19935763 | G | A | 0.87 | 0.063 | 0.009 | 1.11E-11 | 61,111 | 8.98E-04 | 54.91 |
| rs571312 | 18 | 57839769 | A | C | 0.23 | 0.052 | 0.007 | 8.80E-13 | 61,111 | 9.58E-04 | 58.58 |
| rs76227980 | 18 | 58036384 | C | T | 0.98 | 0.14 | 0.023 | 8.68E-10 | 61,111 | 7.68E-04 | 46.99 |
| rs184566112 | 18 | 55943926 | A | T | 0.84 | 0.057 | 0.011 | 4.24E-08 | 61,111 | 8.73E-04 | 53.42 |

Chr, chromosome; EA, effect allele; OA, other allele; EAF, effect allele frequency.

**Table S3. Effect estimates of the associations of genetic instrumental variables for Childhood BMI and risk of lung cancer.**

|  | **No.SNP** | **IVW** | | **MR-Egger** | | **Weighted Median** | | **MR PRESSO**  **(Outlier-corrected)** | |
| --- | --- | --- | --- | --- | --- | --- | --- | --- | --- |
|  |  | **OR (95% CI)** | ***P value*** | **OR (95% CI)** | ***P value*** | **OR (95% CI)** | ***p value*** | **OR (95% CI)** | ***P value*** |
| ***Childhood BMI on lung cancer*** | | | | | | | | | |
| ILCCO | 20 | 0.99 (0.83-1.17) | 0.868 | 0.91 (0.52-1.58) | 0.737 | 0.95 (0.81-1.13) | 0.596 | 0.97 (0.83-1.12) | 0.674 |
| FinnGen | 21 | 1.08 (0.88-1.33) | 0.440 | 0.72 (0.38-1.36) | 0.325 | 1.06 (0.81-1.38) | 0.677 | - | - |
| **Meta-analysis^#^** | | 1.03 (0.90-1.17) | 0.705 | 0.82 (0.54-1.25) | 0.361 | 0.98 (0.85-1.13) | 0.776 | - | - |
| ***Childhood BMI on lung adenocarcinoma*** | | | | | | | | | |
| ILCCO | 19 | 0.95 (0.82-1.11) | 0.519 | 0.85 (0.54-1.34) | 0.487 | 0.96 (0.77-1.19) | 0.698 | - | - |
| FinnGen | 21 | 1.18 (0.84-1.65) | 0.333 | 0.83 (0.28-2.44) | 0.735 | 1.01 (0.63-1.62) | 0.952 | - | - |
| **Meta-analysis** | | 0.99 (0.86-1.13) | 0.832 | 0.85 (0.56-1.29) | 0.437 | 0.97 (0.79-1.18) | 0.752 | - | - |
| ***Childhood BMI on lung squamous cell carcinoma*** | | | | | | | | | |
| ILCCO | 19 | 0.95 (0.82-1.11) | 0.519 | 0.85 (0.54-1.34) | 0.487 | 0.96 (0.78-1.18) | 0.688 | - | - |
| FinnGen | 21 | 1.22 (0.78-1.91) | 0.379 | 0.83 (0.19-3.54) | 0.799 | 0.98 (0.58-1.65) | 0.942 | - | - |
| **Meta-analysis** | | 0.97 (0.84-1.13) | 0.726 | 0.85 (0.55-1.31) | 0.457 | 0.96 (0.79-1.17) | 0.699 | - | - |
| ***Childhood BMI on small cell lung cancer*** | | | | | | | | | |
| ILCCO | 17 | 1.12 (0.80-1.56) | 0.516 | 0.99 (0.33-2.96) | 0.992 | 1.06 (0.72-1.56) | 0.761 | 1.09 (0.80-1.48) | 0.595 |
| FinnGen | 21 | 1.01 (0.57-1.78) | 0.986 | 0.54 (0.09-3.41) | 0.523 | 1.05 (0.52-2.11) | 0.898 | - | - |
| **Meta-analysis** | | 1.09 (0.82-1.45) | 0.554 | 0.84 (0.33-2.15) | 0.720 | 1.06 (0.75-1.48) | 0.746 | - | - |
| ***Childhood BMI on lung cancer in ever smoker*** | | | | | | | | | |
| ILCCO | 21 | 0.90 (0.74-1.11) | 0.329 | 0.73 (0.38-1.41) | 0.356 | 0.89 (0.72-1.09) | 0.262 | 0.97 (0.80-1.17) | 0.763 |
| ***Childhood BMI on lung cancer in never smoker*** | | | | | | | | | |
| ILCCO | 21 | 0.93 (0.68-1.28) | 0.674 | 0.85 (0.31-2.37) | 0.764 | 1.08 (0.71-1.66) | 0.712 | - | - |

Note: ^#^results of meta-analyses were from fixed-effect model.

IVW: Inverse variance weighted; MR PRESSO: MR Pleiotropy Residual Sum and Outlier; OR: Odds Ratio; CI: Confidence Interval.

**Table S4. Heterogeneity and MR-Egger regression analysis of the associations between childhood BMI and lung cancer susceptibility.**

| **Variable** | **Heterogeneity P** | |  | **MR-Egger regression** | |
| --- | --- | --- | --- | --- | --- |
|  | **MR-Egger** | **IVW** |  | **Intercept (SE)** | **Intercept P** |
| ***ILCCO*** | | | | | |
| Lung cancer | <0.001 | <0.001 |  | 0.0052 (0.0171) | 0.764 |
| Lung adenocarcinoma | 0.607 | 0.654 |  | 0.0074 (0.0140) | 0.606 |
| Squamous cell lung cancer | 0.607 | 0.654 |  | 0.0074 (0.0141) | 0.606 |
| Small cell lung cancer | 0.082 | 0.110 |  | 0.0076 (0.0344) | 0.828 |
| Lung cancer in ever smoker | <0.001 | <0.001 |  | 0.0140 (0.0206) | 0.506 |
| Lung cancer in never smoker | 0.305 | 0.361 |  | 0.0059 (0.0321) | 0.855 |
| ***FinnGen*** | | | | | |
| Lung cancer | 0.143 | 0.112 |  | 0.0261 (0.0197) | 0.201 |
| Lung adenocarcinoma | 0.353 | 0.385 |  | 0.0227 (0.0335) | 0.507 |
| Lung squamous cell carcinoma | 0.053 | 0.058 |  | 0.0252 (0.0453) | 0.584 |
| Small cell lung cancer | 0.132 | 0.118 |  | 0.0395 (0.0571) | 0.498 |

IVW: Inverse variance weighted; SE: standard error.

**Table S5. Causal effect of childhood BMI on risk factors of lung cancer.**

| **Variable** | **No.SNP** | **0R (95% CI)** | ***P value*** |
| --- | --- | --- | --- |
| Age of smoking initiation | 21 | 1.01 (0.96-1.06) | 0.817 |
| Smoking: ever vs. current | 21 | 0.99 (0.96-1.02) | 0.527 |
| Smoking: ever vs. never | 21 | 0.94 (0.90-0.98) | 0.006 |
| Cigarettes smoked per day | 21 | 0.94 (0.90-0.98) | 0.003 |
| Drinking per week | 21 | 1.03 (0.98-1.09) | 0.276 |
| Years of education | 20 | 0.99 (0.96-1.02) | 0.600 |
| Total cholesterol | 12 | 0.95 (0.88-1.02) | 0.180 |

OR: Odds Ratio; CI: Confidence Interval.
